# Reduction in deep sternal wound infection with use of a peristernal cable-tie closure system: a retrospective case series

**DOI:** 10.1186/s13019-015-0378-7

**Published:** 2015-11-14

**Authors:** Meghan M. Stelly, Charles B. Rodning, Terry C. Stelly

**Affiliations:** Clemson University, Clemson, South Carolina USA; Department of Surgery, College of Medicine and Medical Center, University of South Alabama, Mobile, Alabama USA; Cardiothoracic and Vascular Surgical Associates, 1855 Springhill Avenue, Mobile, 36607 Alabama USA

**Keywords:** Sternum, Infection, Sternum, Surgery, Devices, Surgical equipment

## Abstract

**Background:**

Deep sternal wound infections are a rare but serious complication after median sternotomy. We evaluated the incidence of deep sternal wound infection associated with two techniques for sternal closure.

**Methods:**

In this retrospective case series, we recorded the method of sternal closure in consecutive patients undergoing a variety of cardiothoracic surgical procedures. Sternal closure in the historical control group was performed using trans-sternal, stainless-steel wire sutures; subsequent patients were closed using wire sutures in conjunction with a novel, peristernal cable-tie closure system to reinforce the *corpus sterni*. Perioperative care was standardized between groups. Demographics, risk factors, and postoperative outcomes were analyzed.

**Results:**

Between July 2010 and July 2014, 609 consecutive adult patients underwent sternal closure following open median sternotomy at a single hospital in Mobile, Alabama. Sternal closure was accomplished with wire sutures in the first 309 patients and with cable-tie reinforcement in the subsequent 300 patients. Baseline characteristics were comparable between groups, except that the cable-tie group exhibited greater preoperative comorbidity. Mean body mass index was comparable between groups (30.2 ± 6.6 kg/m^2^ wire suture versus 30.5 ± 7.7 cable-tie, *p* = 0.568). Deep sternal wound infection occurred in 2.6 % (8/309) patients in the wire-suture group, whereas no deep sternal wound infections were observed in the cable tie group (*p* = 0.008).

**Conclusions:**

The peristernal cable-tie system was a simple and reliable method for sternal closure after open median sternotomy, and was associated with a reduced risk of deep sternal wound infection, even in an obese and comorbid population.

## Background

Despite the increasing use of minimally invasive approaches, the majority of cardiac operations are still performed by way of a median sternotomy [1]. In our practice, this is the most common operative approach for adult cardiac procedures because it is easily and expeditiously performed and provides excellent exposure.

Although post-sternotomy complications are rare, the occurrence of a deep sternal wound infection (DSWI) can be catastrophic, with reported mortality rates between 19 and 29 % [2]. Despite modern prophylactic measures, the reported incidence of DSWI persists at between 0.8 and 6.0 % [3–8].

DSWI is associated with multiple risk factors, including obesity, pulmonary disease, diabetes mellitus, renal failure, advanced age, malnutrition, osteoporosis, and bilateral internal thoracic arterial harvest [3, 6, 9–15]. Postoperative complications, such as the low cardiac output syndrome, respiratory failure, and re-exploration for postoperative hemorrhage, have also been shown to contribute to higher incidence of DSWI [16–19]. Effective strategies for reducing the risk of DSWI include appropriately timed and dosed administration of intravenous antibiotic medications, optimal aseptic/antiseptic skin preparation, avoidance of shaving, and intranasal administration of mupricion [10, 20–22]. Intensive perioperative glucose control has also been proposed to decrease DSWI in patients undergoing coronary artery bypass (CABG) procedures, although the precise targets and appropriate subpopulations remain a matter of debate [13].

Another potential risk factor for DSWI is sternal wound instability, which has been reported to compromise wound integrity and predispose patients to infection [23–25]. However, the relationship between this instability, DSWI, and emerging methods for sternal closure after median sternotomy remains to be explored.

Our practice has historically relied on the placement of a series of parasternal stainless steel wire sutures to coapt the sternum at multiple points from the *manubrium sterni* to the most caudal portion of the *corpus sterni*. In 2012, we adopted the use of a novel, polyether ether ketone (PEEK) based, parasternal cable-tie closure system to replace the wire-suture closure at the *corpus sterni*. This portion of the sternum has been shown to be more fragile and more subject to potentially disruptive forces during patient recovery than the more cephalad portions of the sternum [1]. We hypothesized that the presence of the wide cable ties would help to stabilize the *corpus sterni* during healing, reducing the risk of DSWI. The present retrospective chart review was designed to determine whether the change in our technique affected the incidence of DSWI in our practice.

## Methods

The protocol for this retrospective chart review was approved by the institutional review board at Springhill Medical Center (Mobile, Alabama). Individual informed consent was waived.

### Patients and data collection

Hospital and office charts were reviewed for all consecutive adult patients who underwent sternal closure after primary or repeat median sternotomy for cardiothoracic procedures at Springhill Medical Center during the study period. Data regarding demographics, risk factors, intraoperative variables, sternal closure method, and postoperative outcomes were recorded.

### Patient preparation and perioperative antibiotic medications

All patients received a shower with 2 % chlorhexidine gluconate (CareFusion Corporation, San Diego, CA), along with 2 % mupirocin ointment to both nostrils, on the evening prior to and the morning of the operation. Removal of excess hair from sternum, groin, and bilateral hind limbs (circumferential) was performed with clippers in the operating room. Chlorhexidine gluconate (2 % in 70 % isopropyl alcohol; ChloraPrep, CareFusion Corporation) was applied topically as a skin disinfectant. Bio drapes (Medline Industries, Inc., Mundelein, IL) and disposable paper drapes were then applied sequentially.

All patients received perioperative antibiotic medications according to the following protocol: All patients received intravenous cefazolin timed to be completed 30 minutes prior to skin incision (2.0 g for less than 80 kg body weight, 3.0 g for 60 to 109 kg, and 3.5 g for 110 to 124 kg, 4.0 g for greater than 124 kg.) An additional 1.0 gram was given just prior to initiation of cardiopulmonary bypass, and one half the loading dose was given every 3 hours thereafter while on bypass. Off-pump cases or bypass cases lasting less than 3 hours did not receive additional antibiotic. In the case of penicillin allergy, patients with preoperative hospital stays of greater than 3 days or patients undergoing valve procedures received a weight-based intravenous vancomycin protocol (1.0 g for less than 80 kg body weight, 1.25 g for 80 to 99 kg, 1.5 g for 100 or more kg) with gentamycin (4.0 mg/kg) single dose, timed to be completed 30 min prior to skin incision. If surgery was delayed for more than 2 hours, an additional single dose of levofloxacin (500 mg) was administered. Patients were re-dosed with one half the original Vancomycin dose at cessation of bypass. Mupirocin was administered to the nares of all patients before leaving the operating room.

### Surgical technique

**Fig. 1:**
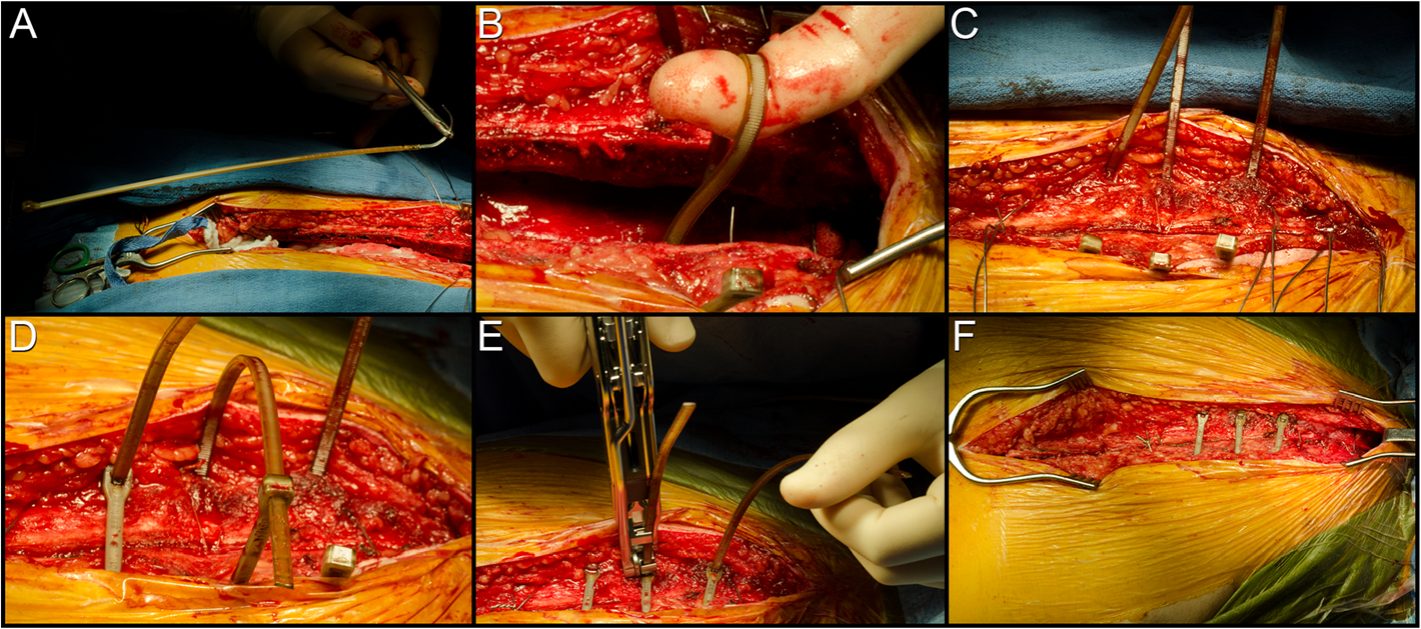
Sternal closure with the hybrid wire-cable-tie method. Following cardiothoracic surgical procedures, the *manubrium sterni* and the caudal end of the *corpus sterni* are coapted with stainless steel monofilament wire suture. Three sterile PEEK-based cable ties are then brought into the field (A) and placed evenly around the medial and cephalad portions of the *corpus sterni* (B-C). The cable ties are tightened (D) and the excess material trimmed (E). The final, fixated sternum is shown in (F)

Median sternotomy was performed in the conventional fashion. In all patients, after completion of the cardiac procedures and achievement of hemostasis, three #7 316 L stainless steel monofilament wire sutures (Ethicon Inc., Somerville, NJ) were placed to coapt the *manubrium sterni* and one #7 316 L stainless steel wire suture was placed to coapt the caudal most portion of the *corpus sterni*. In the wire-suture control group, additional #7 316 L stainless steel monofilament wire sutures were utilized to coapt the cephalad- and mid-portions of the *corpus sterni*. In the cable-tie group, the cephalad- and mid-portions of the *corpus sterni* were coapted with three peristernal cable-tie devices (ZipFix, DePuy Synthes GmbH, Oberdorf, Switzerland) at intercostal levels 3, 4, and 5 (Fig. 1). The presternal fascia and muscle were reapproximated with size 0 polygalactin 910 suture (Vicryl, Ethicon, Inc., Somerville, NJ) applied in a continuous fashion. The subcutaneous tissue was reapproximated with 2–0 polygalactin 910 suture (Vicryl, Ethicon, Inc.) and the skin was closed with a subcuticular 3–0 poliglecaprone 25 suture (Monocryl, Ethicon, Inc.), both applied in continuous fashion in all patients. A sterile island-type bandage was then applied.

### Postoperative follow-up and diagnosis of DSWI

Postoperative care was according to each patient’s primary indication for surgery. After discharge all patients returned for follow-up at 2 weeks, 2 months, and quarterly thereafter. DSWI was diagnosed according to US Centers for Disease Control and Prevention criteria [21]. Any patient exhibiting symptoms of DSWI underwent computed tomography imaging of the chest and plastic surgery consultation. The wounds were managed by formal sternal debridement and placement of a negative pressure therapy device (V.A.C, KCI, Inc, San Antonio, TX), with subsequent muscle flap closure once the septic process was controlled.

### Data analysis

Body mass index was calculated from height to weight data. Patient data were analyzed using SAS software, version 9.2.2. Data are expressed as mean ± standard deviation, n/N (%), or median (minimum-maximum) unless otherwise noted. Significance testing was performed by an independent biostatistician, using a Two-Sample T-test for comparisons between groups (continuous data), either a Chi-square test or a Fisher’s exact test for homogeneity between groups (categorical data), or Wilcoxon Rank-Sum test when median (range) was presented. Alpha was defined as 0.05 for all tests. The primary outcome for this study was the incidence of DSWI in the two groups, and the null hypothesis was that no difference would be observed.

## Results

Between July 2010 and July 2014, sternal incisions were performed on 611 patients at Springhill Medical Center in Mobile, Alabama. Two patients who died intraoperatively were not closed and were therefore excluded from the analysis. A total of 309 patients were closed with the wire suture technique and served as the historical control group, and 300 were closed with the cable-tie system. One of the authors (TCS) performed 71.8 % (222/309) of the wire suture procedures and 92.7 % (278/300) of the cable-tie procedures; the others were performed by a single other partner in his practice. No patients were lost to follow-up. Follow-up evaluation ranged from 21 days to 4 years 5 months, with a median follow-up of 26.6 months.

**Fig. 2:**
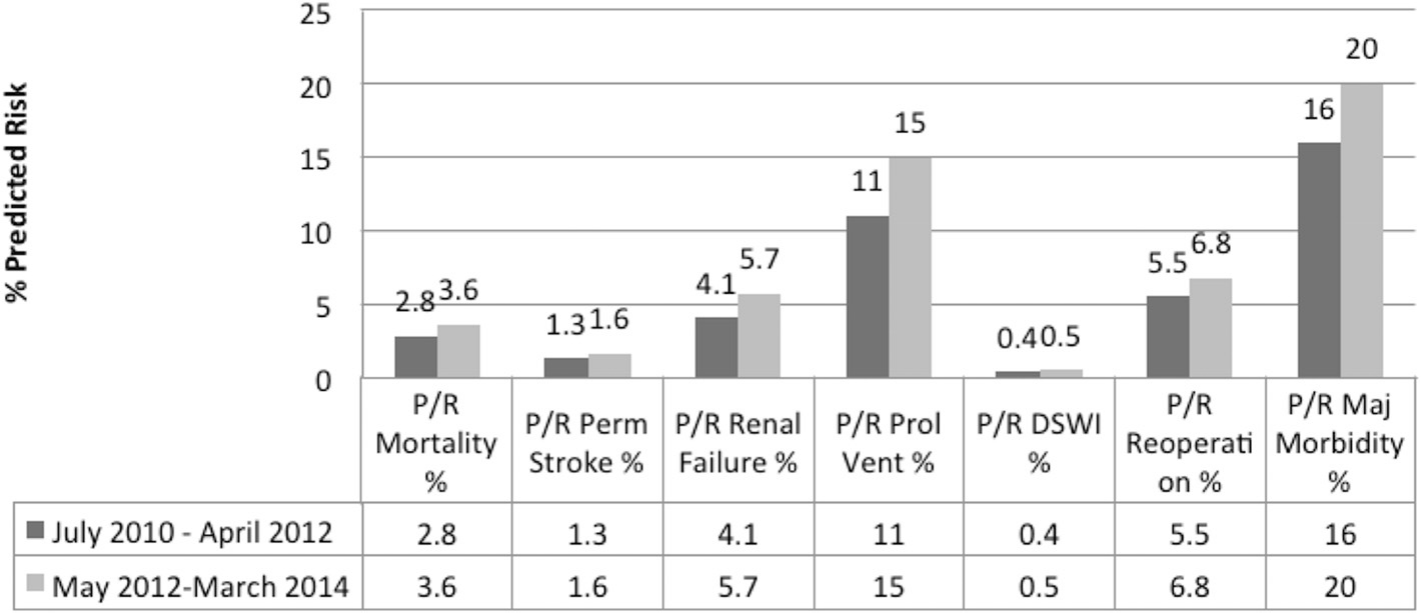
Comparison of STS predicted risk for selected measures

Patient demographics and preoperative risk factors are summarized in Table 1. Age, sex, and average BMI were similar between the two groups and indicative of a generally obese population. Rates of primary and repeat sternotomy were similar between groups. Significantly more patients in the cable-tie closure group were positive for certain risk factors associated with DSWI, including peripheral vascular disease. An analysis of predicted risk scores for this population, using data from the Society of Thoracic Surgeons database, was consistent with these findings and suggested that the cable-tie closure group was somewhat more predisposed to relevant perioperative risks than the standard wire suture group (Fig. 2).

**Table 1:** Summary of baseline and preoperative characteristics for 609 patients undergoing standard median sternotomy^a^

	Total population (*N* = 609)	Standard wire suture (*N* = 309)	Cable-tie closure (*N* = 300)	*p* value
Age, y	64.8 ± 11.4	64.3 ± 10.8	65.3 ± 11.9	0.275
BMI, kg/m^2^	30.4 ± 7.2	30.2 ± 6.6	30.5 ± 7.7	0.608
Sex, male	385 (63.2)	205 (66.3)	180 (60.0)	0.105
Risk factors				
Diabetes	228 (37.4)	107 (34.6)	121 (40.3)	0.146
COPD	118 (19.4)	49 (15.9)	69 (23.0)	0.026*
HTN	505 (82.9)	253 (81.9)	252 (84.0)	0.486
Smoking history	326 (53.5)	162 (52.4)	164 (54.7)	0.580
CHF	162 (26.6)	61 (19.7)	101 (33.7)	<0.001*
PVD	92 (15.1)	34 (11.0)	58 (19.3)	0.004*
Endocarditis	8 (1.3)	2 (0.6)	6 (2.0)	0.171
Renal disease	95 (15.6)	44 (14.2)	51 (17.0)	0.348
Osteoporosis	4 (0.7)	2 (0.6)	2 (0.7)	1.000
Resternotomy	80 (13.1)	33 (10.7)	47 (15.7)	0.069

^a^Data are presented as mean ± standard deviation or n (%) unless otherwise noted. Asterisks (*) indicate statistically significant differences. BMI, body mass index; CHF, heart failure; COPD, chronic obstructive pulmonary disease; CPB, cardiopulmonary bypass; HTN, hypertension; PVD, peripheral vascular disease.

Primary indications for surgery included on-pump CABG in 412 patients, off-pump CABG (35 patients), repair or replacement of one or more valves (270 patients), repair of diseased aorta (26 patients), pulmonary embolectomy (10 patients), and other procedures (8 patients). Intraoperative variables are summarized in Table 2.

**Table 2:** Intraoperative variables for 609 patients undergoing standard median sternotomy^a^

	Total population (*N* = 609)	Standard wire suture (*N* = 309)	Cable-tie closure (*N* = 300)	*p* value
Urgency				<0.001*
Elective	481 (79.0)	220 (71.2)	261 (87.0)	
Urgent	11 (1.8)	10 (3.2)	1 (0.3)	
Emergent	117 (19.2)	79 (25.6)	38 (12.7)	
CPB time, min	70.1 (37.3)	69.6 (38.5)	70.7 (36.1)	0.719
Operative time, min	226.8 (56.6)	224.2 (56.3)	229.4 (56.8)	0.258
Tracheostomy	10 (1.6)	3 (1.0)	7 (2.3)	0.217

^a^Data are presented as mean ± standard deviation or n (%) unless otherwise noted. CPB, cardiopulmonary bypass; LAA, left atrial appendage.

### Deep sternal wound infections

The incidence of DSWI in the historical control group was 2.6 % (8 of 309 patients). No DSWI was observed in the experimental group (*p* = 0.008).

### Secondary outcomes and adverse events

There have been 19 late deaths in this patient series; 8 were in the control group and 13 in the implant group for an overall mortality rate of 2.6 % and 4.3 %, respectively. All late deaths were related to native disease and not to wound problems, and mortality rates were consistent with the calculated STS predicted mortality for each group (Fig. 2). Other than DSWI, the rate of adverse events was generally similar between groups (Table 3). Median ventilation times and ICU and hospital lengths of stay were comparable, but the ranges were broader in the cable-tie group, possibly reflecting the more comorbid population.

**Table 3:** Secondary outcomes and adverse events in 609 patients after sternal closure with standard wire suture or cable-tie system^a^

	Total population (*N* = 609)	Standard wire suture (*N* = 309)	Cable-tie closure (*N* = 300)	*p* value
Sternal dehiscence	8 (1.3)	7 (2.3)	1 (0.3)	0.069
Return to operating room	71 (11.7)	41 (13.3)	30 (10.0)	0.209
Re-exploration for bleeding	26 (4.3)	13 (4.2)	13 (4.3)	0.939
Rewiring	14 (2.3)	9 (2.9)	5 (1.7)	0.305
Perioperative MI	2 (0.3)	2 (0.6)	0 (0.0)	0.499
Postoperative blood loss, first 18 hours, L	0.77 (0–7.6)	0.76 (80–7.3)	0.81 (0–7.6)	0.776
Superficial sternal wound infection	17 (2.8)	12 (3.9)	5 (1.7)	0.097
Ventilation time, days	1 (0–70)	1 (0–30)	1 (0–70)	0.060
Length of ICU stay, days	2 (0–57)	2 (0–39)	3 (0–57)	<0.001*
Length of hospital stay, days	8 (1–73)	8 (1–54)	8 (3–73)	0.050*

^a^Data are presented as n (%) or median (min - max) unless otherwise noted. Asterisks (*) indicate statistically significant differences. ICU, intensive care unit; MI, myocardial infarction

## Discussion

Devices that provide rigid or otherwise secure osseous and soft-tissue coaptation and fixation have been shown to promote earlier union and primary healing [26]. These are especially important in the caudal half of the *corpus sterni,* where physiologic forces have been shown to distract disproportionately. Using human cadavers, McGregor and colleagues observed that intrapleural pressure can exceed 300 mmHg during a cough [1], meaning that a force of 150 N (150 kg-m/s^2^) could routinely be experienced across the sternum during normal physiologic conditions. Divided evenly across the standard 6 twisted stainless steel monofilament wire suture closure, this force exceeds the 20 to 22 N associated with wire fatigue, fracture, loosening, or cutting into sternal bone. They also observed that the caudal end of the *corpus sterni* was most vulnerable, as it was thinner than the *manubrium sterni* and lacked clavicular support.

The ideal material for sternal closure and fixation would be simple, inexpensive, reproducible, durable, would provide adequate support to the caudal portion of the *corpus sterni*, and would allow for rapid reentry in the event of emergent re-exploration. While many techniques for sternal fixation have been tried, each has had its limitations, and stainless steel monofilament wire suture remains the current standard of care. For example, rigid-plate fixation has been demonstrated to be structurally superior to wire suture closure, but adds operative time and costs and is problematic during emergent re-entry [26, 27]. Wider configurations of various suture materials have also been tried, based on the hypothesis that a larger suture surface area would allow more favorable distribution of distracting forces and better stabilization compared to wires. Polyethylene terephthalate ribbon, jacketed steel wires, and steel bands have been tried but have not been widely adopted, possibly because of the complexity of application [16, 19, 28, 29]. Nylon bands are associated with an increased incidence of DSWI [30, 31].

The PEEK-based cable ties used in the present study are wider than monofilament wire suture (4.2 mm versus 0.7 mm) and may provide additional support by providing a larger surface area for osseous contact. We found the ties to be simple to deploy and their cost was relatively inexpensive at less than $100 per tie.

In a retrospective, non-inferiority study of sternal closure in 680 patients, the reported incidence of DSWI was 6 % in the total population, and was comparable in both the cable-tie and standard-of-care groups (*n* = 95 and 498, respectively) [32, 33]. While the results showed that the cable tie system adequately stabilized the sternum, the study was not designed or adequately powered to determine superiority. Our study, while not a prospective, randomized trial, does include more balanced distribution of patient groups and was designed to reduce the potentially confounding selection bias that limited the previous studies.

It is of interest to note that our results found lower incidence of DSWI in the cable-tie group, even though both groups had similar BMI scores that were high compared to the national average. Obesity is known to increase the risk of postoperative wound infections, although the exact mechanisms are not well understood and may include hypovascularity of adipose tissue, decreased oxygen tension, compromised collagenization, compromised immunity, oxidative stress, and adiponectin deficiency [34–38]. The cable-tie group was also generally a more comorbid population, which might have been expected to predispose them to DSWI.

### Limitations

This study was limited by its retrospective design and the potential for a time effect since the control group was operated on first. Follow-up times were relatively short for some patients in the cable tie group, but it should be noted that DSWI typically manifests within 2 weeks postoperatively and this is well within the minimum follow-up time reported. Additionally, since wire sutures and cable ties were used together, we are unable to draw conclusions about the efficacy and safety of the cable ties as a standalone method for sternal fixation.

### Conclusion

We conclude that the cable-tie closure system, when used in conjunction with wire sutures, is a simple and reliable method for coapting the *corpus sterni* after median sternotomy. Our results also suggest that use of the cable ties for this purpose may reduce the risk of DSWI compared to wire sutures alone.
